# Correlation between Angiotensin Serum Levels and Very-Low-Frequency Spectral Power of Heart Rate Variability during Hemodialysis

**DOI:** 10.3390/life12071020

**Published:** 2022-07-09

**Authors:** Leonardo del Valle-Mondragón, Brayans Becerra-Luna, Raúl Cartas-Rosado, Oscar Infante, Héctor Pérez-Grovas, Larissa I. Lima-Zapata, Claudia Lerma, José Rodríguez-Chagolla, Raúl Martínez-Memije

**Affiliations:** 1 Departamento de Farmacología, Instituto Nacional de Cardiología Ignacio Chávez, Juan Badiano 1, Mexico City 14080, Mexico; leonardodvm65@hotmail.com; 2 Departamento de Instrumentación Electromecánica, Instituto Nacional de Cardiología Ignacio Chávez, Juan Badiano 1, Mexico City 14080, Mexico; pacorro28144@gmail.com (B.B.-L.); osinfa@yahoo.com (O.I.); larissalimazap94@gmail.com (L.I.L.-Z.); dr.claudialerma@gmail.com (C.L.); 3 School of Biomedical Engineering, Universidad Anáhuac México, Avenida Universidad Anáhuac 46, Col. Lomas Anáhuac, Huixquilucan 52786, Estado de Mexico, Mexico; rcartas@gmail.com; 4 Departamento de Nefrología, Instituto Nacional de Cardiología Ignacio Chávez, Juan Badiano 1, Mexico City 14080, Mexico; hpgrovas@gmail.com; 5 Departamento de Nefrología, Centro Médico ISSEMYM Arturo Montiel Rojas, Toluca City 52170, Mexico; jm.rodriguez.chagolla@gmail.com

**Keywords:** angiotensin II, angiotensin 1–7, autonomic cardiac modulation, end-stage renal disease, hemodialysis

## Abstract

Cardiovascular regulatory mechanisms that fail to compensate for ultrafiltration and cause hypovolemia during hemodialysis (HD) are not completely understood. This includes the interaction between the autonomic nervous system and the biochemistry that regulates blood pressure and modulates cardiac activity and vascular tone in response to hypovolemia in patients treated with HD. The objective was to evaluate the association of spectral indices of heart rate variability (HRV) with serum levels of angiotensin II, angiotensin 1–7, nitric oxide and total antioxidant capacity during HD. Electrocardiographic records were obtained from 20 patients during HD (3 h), from which HRV data and spectral power data in the very-low-frequency (VLF), low-frequency (LF) and high-frequency (HF) bands were generated. Three blood samples per patient were collected during HD (0.0, 1.5, 3.0 h) to determine the levels of biomarkers involved in the pressor response during HD. Angiotensin II had a positive correlation with VLF (r = 0.390) and with LF/HF (r = 0.359) and a negative correlation with LF (r = −0.262) and HF (r = −0.383). There were no significant correlations between HRV and the other biomarkers. These results suggest that during HD, VLF could reflect the serum levels of angiotensin II, which may be associated with the autonomic response to HD.

## 1. Introduction

Hemodialysis (HD) benefits more than 2 million end-stage renal disease (ESRD) patients worldwide [1] by removing uremic toxins and correcting abnormal liquid accumulation, electrolyte concentration and acid–base equilibrium. This is performed over a short period (i.e., between 3 and 4 h), which can induce fast or abrupt changes, increasing the risk of hemodynamic instability and subsequent intradialysis hypotension or hypertension [2].

HD causes hemodynamic, thermal and respiratory stress, which produce adverse effects in the patient, both in the medium and long term [3]. Hemodynamic stress causes a decrease in both blood volume and blood pressure in the circulatory system, which together can cause intradialytic hypotension and reduced tissue perfusion, damaging vital organs, such as the heart and the brain, in addition to generating a deformation of the vascular bed due to the sudden pressure changes inherent to the HD process, favoring the appearance of biochemical alterations related to systemic vasoconstriction and vasorelaxation [3]. HD therapy increases the body’s core temperature, which leads to thermal stress and subsequent vasodilation in the skin, to redistribute blood volume and dissipate excess accumulated heat. This action counteracts the normal response to hypovolemia and contributes to the occurrence of adverse events [3]. Finally, respiratory stress induces prolonged arterial hypoxemia, which increases the severity of tissue oxygenation [3].

For its part, the renin angiotensin aldosterone system (RAAS) is one of the most important mechanisms that regulates systemic blood pressure and renal hemodynamics; its dysregulation promotes both sodium and water retention and the incidence of arterial hypertension [4,5]. The RAAS is composed of a series of interactions between enzymes and substrates that generate peptide hormones with critical functions in cardiovascular regulation [6]. The autonomic nervous system (ANS) also interacts with the RAAS to carry out cardiovascular regulation [5]. Thus, within RAAS, angiotensin II has its main action on type I receptors, which has a vasopressor effect that includes increased tone of the sympathetic nervous system (SNS), decreased tone of the parasympathetic nervous system (PNS) and baroreflex sensitivity, which increases systemic blood pressure, vasoconstriction and aldosterone levels, causing an increase in sodium and the consequent fluid retention [5]. On the other hand, RAAS opposes the actions of angiotensin II, producing angiotensin 1–7, which lowers systemic blood pressure by decreasing the tone of the SNS and increasing the tone of the PNS along with the baroreflex sensitivity [5,6]. In addition to its vasodilator effect, angiotensin 1–7 interacts biochemically with endogenous modulation systems to produce nitric oxide and vasodilation when coupled with its G protein-coupled receptor known as MAS [7,8]. Thus, angiotensin II and angiotensin 1–7 are the main modulators of the balance of systemic blood pressure. The nitric oxide activated by angiotensin 1–7 acts as an effector molecule on all vascular beds that activate second messengers involved in hemodynamic regulation, this being dependent on shear stress and pressure deformation in the vascular bed, activating the SNS and the PNS.

**Figure 1:**
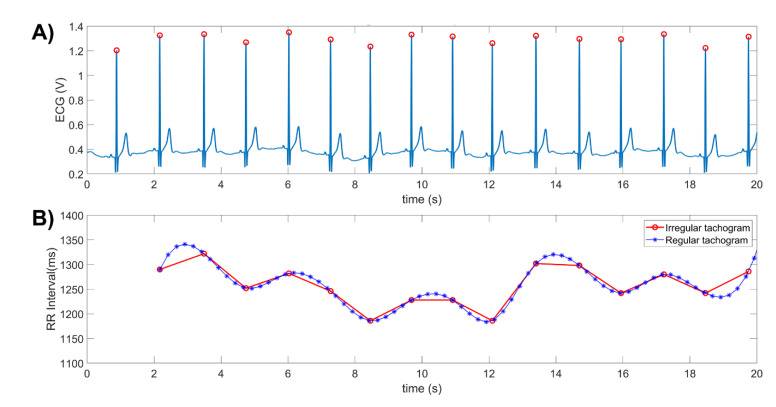
(**A**) 20 s trace of an ECG recording that was conditioned for the detection of R waves, with the detected R waves shown in red circles. (**B**) RR intervals (time intervals between the R waves), which had irregular time sampling periods (red trace). The original tachogram (red trace) must be resampled at regular time intervals (blue trace) before the application of several spectral analysis methods.

The effects of the RAAS on the activity of the ANS are reflected in the modulation of the sympathetic and parasympathetic flows that regulate the cardiovascular system [5]. The modulation of the ANS on the pacemaker of the heart (sinus node) causes an increase or decrease in the time that elapses between consecutive beats [9,10]. This variation in the time interval between consecutive R waves is known as heart rate variability (HRV) (Figure 1), which can be evaluated using analysis techniques in the time and frequency domains [10]. Heart rate (HR) and HRV are inversely proportional as are the modulatory effects of the SNS and PNS. While sympathetic outflow increases the heart rate and decreases HRV, parasympathetic outflow decreases the heart rate and increases HRV.

**Figure 2:**
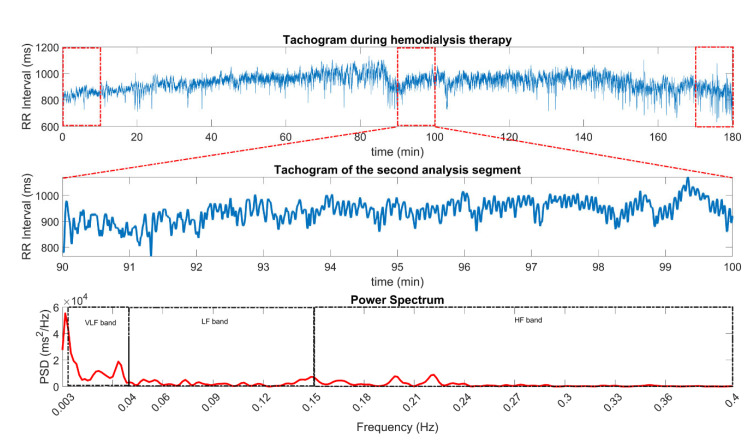
Heart rate variability (HRV) time series, also known as RR intervals, from an entire HD session, where the selected 10 min segments for further analysis are highlighted with dashed, red boxes (**upper panel**). The segment corresponding to 1.5 h after the beginning of HD (second segment) is shown in the **middle panel**, with the corresponding power spectrum density (PSD) plot is in the **lower panel**.

The most common method to obtain HRV is through an electrocardiographic (ECG) recording, from which the time intervals between consecutive R waves are measured to form a time series called the HRV recording or R-R tachogram (Figure 1). HRV assessment can be performed using time domain [10] or frequency domain analysis techniques [10,11,12,13,14,15,16,17,18,19,20,21]. Spectral analysis of HRV is accepted as an indirect method to assess interactions between the ANS and the cardiovascular system. To perform this assessment, the total power spectrum of the HRV recording must be estimated and divided into three or four bands for analysis, depending on the length of the record and the objectives to be achieved (Figure 2). The spectral power of HRV that is within the 0–0.4 Hz range is divided into high-frequency (HF: 0.15–0.4 Hz), low-frequency (LF: 0.04–0.15 Hz) and very-low-frequency (VLF: 0.003–0.04 Hz) [10]. The power indices obtained from these bands are associated with different physiological activities [10,21]. HF is related to PNS activity and is also influenced by the respiratory rate; LF is associated with both SNS and PNS activities as well as the baroreflex activity; VLF is associated to hormonal, vasomotor, thermoregulatory and RAAS influences [10,21].

Recent studies on the cardiovascular control in ESRD patients showed that VLF power increases during hemodialysis [16] and that such an increase in VLF is different in patients who maintain stable blood pressure compared to patients who develop intradialytic hypotension [15,19]. These results suggest that the RAAS may have a significant role in cardiovascular control to prevent sudden drops in systemic blood pressure. Additionally, it was found that in patients who did not present hypotension there was a constant participation of both the SNS and the PNS, although in different proportions.

Considering that the standards for HRV spectral analysis do not allow for estimating the VLF with confidence [10], the correlation between VLF and the serum levels of RAAS markers of ESRD patients during HD therapy has not been studied. Moreover, since there are different mechanisms of bidirectional regulation between the RAAS and the ANS, and the action of both systems is reflected in the variability of the heart rate, we hypothesized that during hemodialysis, the variation in the serum levels of angiotensin II and angiotensin 1–7 are reflected in the VLF spectral index of HRV and that the variation between these biomarkers and power in VLF presents a significant correlation. The aim of this exploratory work was to determine the correlation of the serum levels of some RAAS markers (i.e., angiotensin II, angiotensin 1–7, total antioxidant capacity and nitric oxide) with the spectral indices of HRV (i.e., power in the bands of VLF, LF and HF) during HD.

## 2. Materials and Methods

### 2.1. Patients

This prospective, cross-sectional and quasi-experimental study included a nonrandom sample of 20 ESRD patients treated with HD therapy at the Instituto Nacional de Cardiología Ignacio Chávez. All patients signed written consent to participate in the study protocol, which was approved by the Research and Bioethics Committees of our institution (number: 17-1014). Patients received three sessions of HD per week for at least 6 months prior to the start of the study. The study included patients from both sexes, aged between 21 and 68 years old, who were on dry weight without antihypertensive drug treatment and without decompensated lungs or heart disease. Patients with anatomical or functional limitations that prevented the placement of electrodes for ECG recording or who had vascular access dysfunction were excluded. According to the routinely clinical management, patients did not have dietary restrictions beyond the standardized advice for ESRD patients to avoid excess salt and drinking. Patients usually performed aerobic exercise during HD with an ergonomic bicycle without resistance. None of them had a prescription for erythropoietin. They had a stable hemoglobin within an expected target for ESRD without exogenous erythropoietin most likely by two mechanisms: (i) by a preserved nutritional status with unrestricted protein intake; (ii) by the hypoxia-inducible factor pathway activation due to the high altitude of Mexico City, which is 2240 m above sea level, as the beneficial effect of altitudes above 1000 m has been documented on the hemoglobin levels in ESRD patients [22,23]. 

The general characteristics of the patients, expressed as the mean ± standard deviation, were age = 37 ± 12 years, albumin = 4.1 ± 0.23 g/dL and hemoglobin = 10.5 ± 2.0 g/dL. Three patients had a history of cardiovascular event. The ESRD etiology was immunoglobulin A nephropathy (n = 2), lupus nephritis (n = 3), hypertensive nephrosclerosis (n = 2), membranoproliferative glomerulonephritis (n = 3), autosomal dominant polycystic kidney disease (n = 1), diabetic nephropathy (n = 1) or undetermined etiology (n = 8). Prior to HD, patients had a weight gain of 2 ± 0.6 kg. During the HD sessions in the present study, patients underwent replacement of 19.5 ± 1.4 L, the uric acid reduction was 81.3 ± 4.3%, urea reduction was 76 ± 5.1%, creatinine reduction was 71 ± 6.8%, phosphorus reduction was 48.5 ± 4.4% and Kt/V at the end of 3 h was 1.47 ± 0.5. Eight patients had intradialytic hypotension. Intradialytic hypotension is defined as a symptomatic decrease in either systolic blood pressure ≥ 20 mm Hg or a decrease in mean arterial pressure of 10 mm Hg. The symptoms associated with the blood pressure drop might include abdominal discomfort, yawning, nausea, vomiting, muscle cramps, restlessness, dizziness, fainting or anxiety.

### 2.2. Study Protocol

Although patients did not have dietary restrictions in their routinely clinical management, at the end of the last HD session prior to the HD of the study, dietary recommendations were given to the patients so that their interdialytic weight gain did not exceed 2 kg. For those patients who did not adhere to the recommendations, corrective actions were applied during the sessions to standardize the volume extraction. If the weight gain was greater than the pre-established goal, the HD session was extended to reach the patient’s dry weight; if the weight gain was less than the pre-established goal, then a bolus was injected during the session to reach the 2400 mL of liquid extraction established for the protocol. Patients were also asked to refrain from consuming coffee and tobacco for seven days before the study to obtain viable blood samples for assessment of angiotensin II, angiotensin 1–7, nitric oxide and total antioxidant capacity. To maximize the ANS response, during HD therapy all patients were in a supine position without performing physical activity, cognitive tasks or breathing control. Brachial blood pressure, heart rate, central temperature and peripheral temperature were measured with a mCare 300 vital signs monitor (Spacelabs Healthcare, Snoqualmie, WA, USA). Measurements were taken every 10 min since the beginning of the therapy (0 min) up to the end (180 min). Blood pressure measurements were performed with the oscillometric method. For this study, the measurements considered in the analysis were the ones obtained at the beginning of the HD session, after 1.5 h and after 3 h.

### 2.3. Hemodialysis Prescription

In our routine clinical practice, the hemodialysis prescription (including total ultrafiltration time and blood flow rate) was adjusted to each patient’s needs before each session. Only for the session of this study was the hemodialysis adjusted as follows. All HD sessions were performed in the morning using the same HD machine (model 4008, Fresenius Medical Care, Waltham, MA, USA). A polysulfone filter with an effective surface area of 1.8 m^2^ was used. The blood flow rate ranged between 330 and 430 mL/min, and the flow rate was set to 500 mL/min, with a dialyzing solution at 35 °C. The dialysis solution had the following concentrations: Na^+^ = 138 mmol/L, HCO^−3^ = 32 mmol/L, Ca^+2^ = 2.5 mmol/L, K^+^ = 2 mmol/L, Mg^+2^ = 1 mmol/L, acetate = 3 mmol/L and glucose = 200 mg/dL. The ultrafiltration rate was fixed at 800 mL/h to achieve a total extraction volume of 2400 mL over 3 h. A total of 2500 units of heparin was administered to each patient through the venous line of the HD circuit using the following dose: 1000 units were injected as an initial bolus and the remaining 1500 units in continuous infusion during the 3 h of HD. We analyzed a single dialysis session in each patient, without preference for the weekday (8 patients had the mid-week session and 12 had the session during the first day after the weekend).

### 2.4. Blood Sampling and Serum Markers Measurement

Arterial blood samples of 4 mL were taken during HD therapies to assess cardiovascular serum markers. The samples were extracted from the vascular access of the patients from the arterial line circuit before the dialysis filter at the beginning of the HD session, after 1.5 h and after 3 h. The tubes to collect the samples did not have anticoagulant or precipitating gel. During the collection, the blood was poured slowly through the inner wall of the tubes, and during their transportation to the laboratory, they were not shaken to avoid a high degree of hemolysis of the samples.

#### 2.4.1. Angiotensin II and Angiotensin 1–7 Measurement

Angiotensin II and angiotensin 1–7 were determined simultaneously in all samples by capillary zone electrophoresis, according to the methodology of Tenorio LFA et al. (2010) [24]. The samples were deproteinized with methanol (Sigma, St. Louis, MI, USA) in a 1:1 ratio and centrifuged at 16,000× *g* for 15 min at 4 °C (Sorvall RC-28S, SS34 rotor, DuPont, Newtown, CT, USA). It was filtered with 0.22 mm nitrocellulose membrane filters (Millipore, Billerica, MA, USA), diluted 1:10 with 0.1 M sodium hydroxide (Sigma, St. Louis, MI, USA) and analyzed directly. For this purpose, the P/ACETM MDQ system (Beckman Coulter, Urbana, IL, USA) was used, to which the capillary was preconditioned (fused silica capillary with polyamide coating of 60 cm × 80 microns i.d.; Polymicro Technologies, Phoenix, AZ, USA) passing a 0.1 M solution of sodium hydroxide (Sigma, St. Louis, MI, USA) for 10 min, followed by deionized water (Hycel Reactivos Químicos, SA de CV, Zapopan, Jalisco, México) for 10 min, methanol (Sigma, St. Louis, MI, USA) for 10 min and, finally, the running buffer (100 mM boric acid + 3 mM tartaric acid at pH 9.8; Sigma, St. Louis, MI, USA) for 10 min. The samples were injected under hydrodynamic pressure at 0.5 psi/10 s. Separation was performed at 30 kV for 10 min at 200 nm and 10 °C. The migration time for the angiotensin II was 3.329 ± 0.093 min, and for angiotensin 1–7 it was 5.342 ± 0.112 min. The capillary was washed between runs with 0.1 M NaOH (Sigma, St. Louis, MI, USA) for 2 min, methanol (Sigma, St. Louis, MI, USA) for 2 min, deionized water (Hycel Chemical Reagents, SA de CV, Zapopan, Jalisco, Mexico) for 2 min and running buffer for 4 min. The results are expressed in pmol/mL.

#### 2.4.2. Nitric Oxide Measurement

The nitric oxide was quantified indirectly by the method of Tenorio LFA et al. (2005) [25], which is based on the Griess diazotization reaction through the formation of diazonium salts catalyzed by vanadium (V^+3^) instead of cadmium (Cd^+2^). The quantification of nitric oxide was carried out indirectly on the nitrites derived from its degradation, generating a bright green complex, when the diazonium product (diazonium salt + N-(1-naphthyl)-ethylenediamine) was coupled with the V^+3^. This colorful compound turned out to be more stable and less toxic than that generated when Cd^+2^ is used. Thus, 100 µL of vanadium (III) chloride solution (Sigma, St. Louis, MO, USA) at 0.8% (*p*/*v*) in phosphoric acid (Sigma, St. Louis, MO, USA) at 1 M was used. It was manually homogenized and 50 mL of the 2% (*p*/*v*) sulfanilamide solution (Sigma, St. Louis, MO, USA) in phosphoric acid (Sigma, St. Louis, MO, USA) at 5% (*v*/*v*). It was manually homogenized and 50 µL of N-(1-naphthyl) ethylenediamine dihydrochloride solution (Sigma, St. Louis, MO, USA) at 0.2% (*p*/*v*) in deionized water (Hycel Reactivos Químicos, SA de CV, Zapopan, Jalisco, Mexico). It was manually homogenized and allowed to incubate at room temperature for 45 min, protected from light. After, 3 mL of deionized water (Hycel Reactivos Químicos, SA de CV, Zapopan, Jalisco, México) was added, and it was read spectrophotometrically at wavelengths of 572 and 587 nm (UV-1800 Spectrophotometer, Shimadzu Co., Kyoto, Japan), adjusting with a reagent blank. To calculate the nitric oxide concentration, the absorbance at 572 nm was subtracted from the absorbance at 587 nm. The results are expressed in pmol/mL.

#### 2.4.3. Total Antioxidant Capacity Measurement

Total antioxidant capacity was indirectly quantified by the method of Campos C. et al. (2005) [26], which is based on the reducing capacity of plasma to reduce Cu^+2^ to Cu^+1^, due to the effect of circulating endogenous antioxidants. Reduced copper was detected by the formation of stable Cu^+1^ chromogens in the presence of bathocuproine, which are bright light green in color. Thus, 35 µL of plasma was added to 145 µL of 0.1 M phosphate buffer (Sigma, St. Louis, MO, USA) at pH 7.5 and homogenized at 500 rpm (Multi-Vortex V-32, BioSan, Riga, Latvia) for 200 s. Immediately afterwards, 50 µL of 0.01 M copper II chloride (Sigma, St. Louis, MO, USA) was added and homogenized at 500 rpm (Multi-Vortex V-32, BioSan, Riga, Latvia) for 200 s. Next, 50 µL of 0.01 M bathocuproine (Sigma, St. Louis, MO, USA) was added and homogenized again at 500 rpm (Multi-Vortex V-32, BioSan, Riga, Latvia) for 200 s. Finally, the reaction was diluted to 1000 µL with deionized water (Hycel Reactivos Químicos, SA de CV, Zapopan, Jalisco, México) and read spectrophotometrically at a wavelength of 490 nm (UV-1800 Spectrophotometer, Shimadzu Co., Kyoto, Japan), previously adjusting with a phosphate buffer blank. Total antioxidant capacity is expressed in mmol/L.

### 2.5. Electrocardiogram (ECG) Recording and Heart Rate Variability (HRV) Analysis

A 7-lead Holter device (model DMS300-7, DM Systems Co., Ltd., Beijing, China) with a sampling rate of 128 samples per second was used to acquire the ECG in three leads during HD and to obtain consecutive RR intervals (Figure 1). The electrodes were placed according to the Frank lead system (orthogonal configuration), and the acquisition began at least 10 min before starting HD. Recordings were downloaded from the Holter using CardioScan (DM Software Inc., Santa Ana, CA, USA) and processed offline. Of the three channels obtained, the one with the least noise and artifacts was chosen based on a visual inspection of the ECG (Figure 1, upper panel).

The acquired ECG recordings were first resampled with MATLAB (MathWorks Ltd., Natick, MA, USA) at 256 samples per second using linear interpolation to improve the QRS complex detection [27,28]. Then, the resampled ECGs were processed with Kubios HRV analysis software (Kubios Oy, Kuopio, Finland) to identify each R wave peak, to correct false detections, and to measure the time interval between consecutive R waves. This process generates the RR interval time series or tachograms (Figure 2, upper panel), which were subsequently interpolated using a spline method with a sampling rate of 4 samples per second (Figure 1, lower panel). All recordings had fewer than 1% ectopic heart beats. 

The HRV records were processed using the continuous wavelet transform, the bands were separated in the time-scale plane, and then the inverse continuous wavelet transform was applied to extract the spectral components of VLF, LF, and HF [10,16]. Spectral power was estimated in non-overlapping 10 min segments [14] and normalized to unity. Subsequently, the values of the indices of these three bands were selected at the beginning of HD (window of the first 10 min), 1.5 h after HD (window number 9) and at the end of HD (window 18); see Figure 2 (upper panel). The methodology for HRV analysis is described in detail in [16].

### 2.6. Statistical Analysis

The Kolmogorov–Smirnov test was applied to assess whether the variables had a normal distribution, and logarithmic transformation was applied when necessary to achieve a normal distribution. The results are reported as the mean ± standard deviation. Serum angiotensin II, angiotensin 1–7, nitric oxide and total antioxidant capacity were compared during the three sampled times by analysis of variance (ANOVA) with post hoc adjustment of *p*-values using the Bonferroni method. To evaluate the association between the RAAS serum markers and the other study variables, Pearson’s correlation analysis was performed. Linear (stepwise) multiple regression models were used to corroborate the association between HRV indices (as dependent variables) and angiotensin II, age, diastolic blood pressure, heart rate, and central temperature (as independent variables). The statistical analysis was performed with the computer program Statistical Package for Social Sciences (SPSS) version 21.0 (IBM Corp, Armonk, NY, USA). A *p*-value < 0.05 was considered statistically significant. 

## 3. Results

Table 1 shows the values of the study variables separated into the three samples taken during HD as well as the total sample. The average heart rate decreased in the second measurement (1.5 h of HD) compared to the first measurement at the start of HD. VLF in absolute units (ms^2^) increased at the end of HD compared to the first measurement at the start of HD (*p* < 0.05). HF increased in the second measurement when compared to the HF of the first measurement (*p* < 0.05). The other variables had no significant differences during HD.

**Figure 3:**
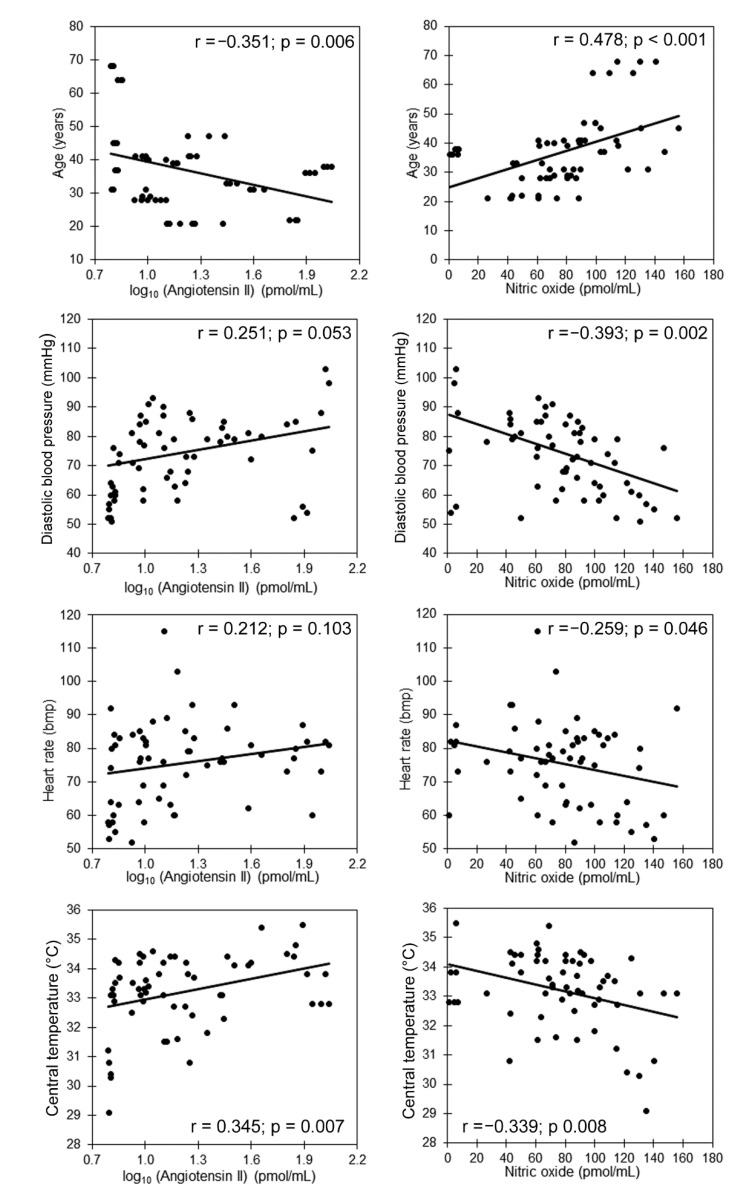
Scatter plots and Pearson’s correlation coefficient (r) of angiotensin II and nitric oxide versus age, hemodynamic variables and core temperature.

**Figure 4:**
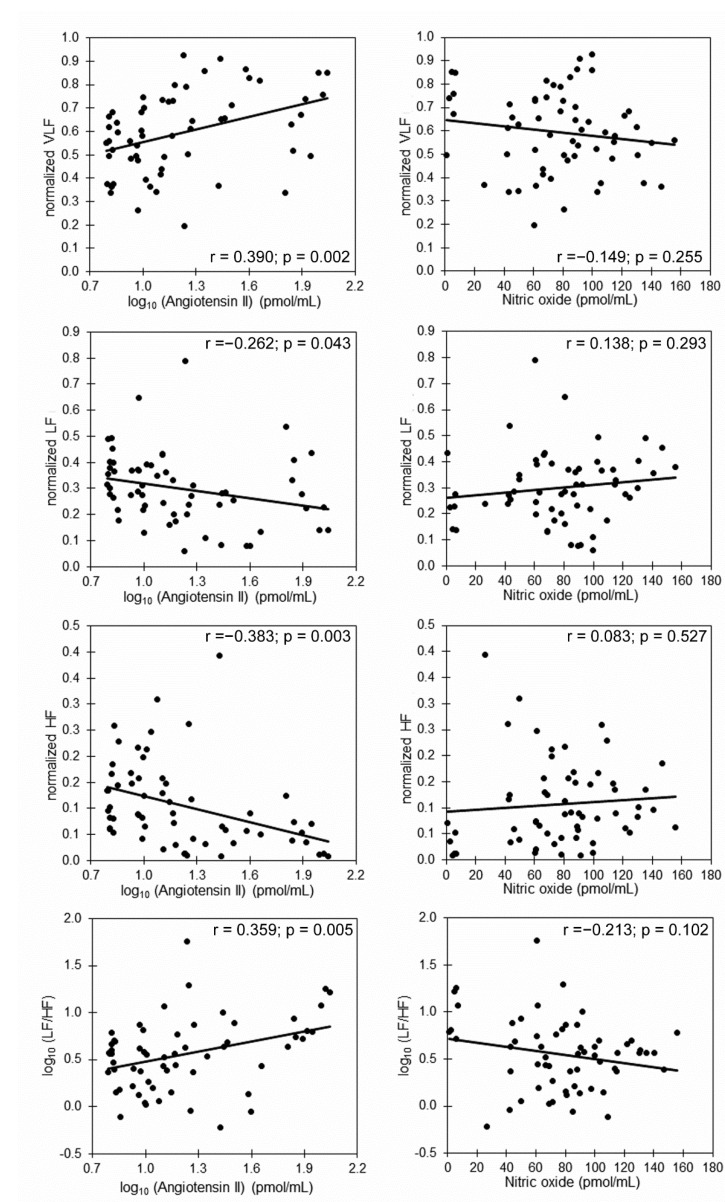
Scatter plots and Pearson’s (r) correlation coefficient of angiotensin II and nitric oxide versus HRV indices.

Considering that there were no significant differences among the three samples for most of the variables (Table 1), we performed the correlation analysis with the total sample (Table 2). Angiotensin II had positive correlations with core temperature and normalized VLF, and it had negative correlations with age, normalized LF, normalized HF and the LF/HF ratio (Table 2, Figure 3 and Figure 4). Nitric oxide was positively correlated with age and negatively correlated with diastolic blood pressure, heart rate and core temperature (Table 2 and Figure 3). Total antioxidant capacity had negative correlations with diastolic blood pressure and core temperature (Figure 2). 

To further explore the association between HRV indices and angiotensin II, a multiple regression analysis was performed. Table 3 shows that when considering age, diastolic blood pressure, heart rate and central temperature as potential confounding (independent) variables, angiotensin II remained significantly associated to normalized VLF, normalized LF, normalized HF and the LF/HF ratio. 

## 4. Discussion 

This study explored the correlation between HRV indices and biochemical parameters during three moments of HD (i.e., 0, 0.5 and 3 h) in a cohort of ESRD patients. Hemodynamic, electrocardiographic and biochemical data were obtained to assess their correlation with markers of the RAAS system (i.e., angiotensin II and angiotensin 1–7) which are involved in the vasodilator response under a stimulating effect of the ANS induced by the hemodynamics’ shear stress.

HD is a renal replacement therapy that provokes hemodynamic, thermic and respiratory stress with adverse consequence for the patient [3]. The resulting change in the cardiovascular homeostasis leads to activation of the RAAS, which interacts with the ANS to compensate for the changes in systemic blood pressure triggered by the depletion of blood volume. The present prospective, transverse study explored the associations between angiotensin II and angiotensin 1–7, nitric oxide and total antioxidant capacity versus HRV spectral indices in the VLF, LF and HF bands. The associations were evaluated by bivariate correlation analysis. The levels of angiotensin II and angiotensin 1–7 were determined by capillary zone electrophoresis, and those of nitric oxide and total antioxidant capacity by UV-Visible spectrophotometry. Spectral indices were determined by applying the wavelet transform to 10 min HRV segments, which is twice the length recommended by the Task Force for HRV spectral analysis [10]. This modification to the standard allows to reduce the uncertainty in the estimation of the spectral power in the VLF band [14]. The results showed an increase in VLF at 3.0 h of HD compared to 0 h, while HF had an increase at 3.0 h compared to 1.5 h of HD. There were no changes during HD in the other HRV indices during HD.

The ESRD patients treated with HD in this study had significant correlations between angiotensin II levels and HRV spectral indices. An increase in angiotensin II during HD was expected as a physiological response to the RAAS activation [3], although we did not identify significant changes in angiotensin II during the three times sampled during HD. Nevertheless, vasoconstriction is needed to increase peripheral resistance in response to HD-induced hypovolemia [29], and such vasoconstriction is initially the result of ANS activation and the activity of vasoactive substances such as angiotensin II [30].

The correlations between angiotensin II and HRV spectral indices were expected due to the bidirectional interactions between angiotensin II and the ANS [5]. The VLF band is the index where the hormonal influences of the RAAS are reflected [21], as well as thermoregulation [3]. Correlations between angiotensin II and LF, HF, and HF/LF spectral ratios suggest that circulating angiotensin II concentration concurs with SNS activation [5]. Other RAAS markers, such as angiotensin 1–7 and nitric oxide, which were also measured, did not show such consistent changes; this may be because they have smaller magnitude effects, or they are attenuated by the action of angiotensin II. However, more studies are required to evaluate such effects.

For the study, the spectrum of the total HRV spectral power was estimated and divided into three bands for analysis: high-frequency (HF: 0.15–0.4 Hz), low-frequency (LF: 0.04–0.15 Hz) and very-low-frequency (VLF: 0.003–0.15 Hz) components. 0.04 Hz) [10]. The power indices obtained from these bands are associated with different physiological activities. HF is related to PNS activity and is also influenced by respiratory rate; LF is associated with the SNS and/or PNS as well as with baroreceptor activity; while the VLF is associated with hormonal, vasomotor, thermoregulatory influences and the RAAS [11,21].

HRV analysis is normally performed in 5 min segments of the signal, according to the standards [10]. Regarding the VLF band, this has been little studied because 5 min of HRV recording are not sufficient to capture it and estimate it with certainty [14]. Previous studies showed that the increase in intradialytic VLF is higher in patients who maintain a stable blood pressure compared to patients who develop intradialytic hypotension [15,19], suggesting the significant role of RAAS to preserve the hemodynamic stability. 

Thus, Table 1 shows the values of the study variables, separated in the three moments (i.e., 0, 1.5 and 3.0 h) of HD and grouped as the total sample. In this case, the average heart rate decreased in the second measurement (1.5 h of HD) compared to the first measurement at the start of HD. VLF in absolute units (ms^2^) increased at the end of HD compared to the first measurement at the start of HD (*p* < 0.05). On the other hand, HF showed an increase in the second measurement when compared to HF of the first measurement (*p* < 0.05). There were no significant differences during HD in the other variables evaluated.

Considering that there was no significant difference in the three samples for most of the variables (Table 1), we performed the correlation analysis with the total sample (Table 2). In this case, the total antioxidant capacity had negative correlations with the diastolic blood pressure and core temperature (Figure 2). Nitric oxide was positively correlated with age, and negatively correlated with diastolic blood pressure, core temperature (*p* < 0.01) and heart rate. Meanwhile, angiotensin II had positive correlations with diastolic blood pressure, core temperature, heart rate, VLF and the LF/HF ratio and had negative correlations with age, LF and HF (Figure 2 and Figure 3).

Ultrafiltration conditions the decrease in effective circulating volume [30], which leads to episodes of transient myocardial ischemia [31], a phenomenon that may be amplified in patients with limited cardiac reserve [32] and/or autonomic dysfunction [33].

The ANS is part of the regulatory mechanisms of blood pressure during HD, promoting the elevation of peripheral vascular resistance and favoring the secretion of hormones that counter regulate arterial tone. Increased vascular resistances lead to reduced flow to the muscular, renal, and skeletal beds, allowing a larger portion of cardiac output to be directed to more critical regional circulations [34].

The importance of RAAS activity during ultrafiltration in HD is controversial. Studies in animal models (dogs with acute nephrectomy) show that the hemodynamic response to bleeding is altered [35]. However, one week after nephrectomy, the hemodynamic response to bleeding or isolated ultrafiltration was well preserved despite the severe decrease in plasma renin activity [36]. On the other hand, HD nephrectomized patients retain a normal hemodynamic response to upright posture [37]. There is a possibility that the RAAS may be important in dialysis patients in whom other sympathetic compensatory responses are diminished. Isolation of the effect of renin is difficult because loss of renin response to HD is often a marker of baroreceptor dysfunction and usually occurs in conjunction with loss of compensatory sympathetic response [38,39]. 

Regarding the use of anti-hypertensives, our routine clinical practice was focused on regulating blood pressure by providing enough hemofiltration and keeping close follow up on the dry weight of each patient. With this approach, we favored the adaptive response of the physiological mechanisms of each patient to hemodialysis. For instance, we showed that despite their augmented chronic sympathetic predominance, patients in hemodialysis have a preserved autonomic capacity to respond to physiological and hemodynamic stimuli [40]. Further studies in patients treated with anti-hypertensives are required to explore the responsiveness of the RAAS system to hemodialysis and the potential impact of the RAAS system interaction with the ANS system, particularly in those treated with ARBs and ACE inhibitors.

In hemodialysis patients, three main reasons for hypertension are volume overload, derangements of the renin–angiotensin system and sympathetic overactivity [41]. Angiotensin II promotes hypertension in these patients through several mechanisms including water retention, vasoconstriction and sympathetic activation [42]. We found that diastolic blood pressure correlated directly with angiotensin II and inversely with nitric oxide. The vasoconstrictor activity of angiotensin II, which induces increased pressure in blood vessels, is largely inhibited by nitric oxide which stimulates vasorelaxation by activating soluble guanylate cyclase which, in turn, activates various signaling pathways dependent on cyclic guanosine monophosphate, inducing an inhibitory effect on renin release, which decreases the production of angiotensin II [43]. Thus, diastolic blood pressure, angiotensin II and nitric oxide have a close biological correlation with the stability of the blood pressure regulatory system. However, we only found significant correlations with diastolic blood pressure and not systolic blood pressure. While systolic blood pressure results from the cardiac output and vessel mechanical properties, diastolic blood pressure, which is coupled with the relaxing or filling cardiac phase of the cardiac cycle, is a consequence of potential energy accumulated in the vessel due to the elastic components of the vessel [44]. In essential hypertension, isolated diastolic hypertension is associated with a different hemodynamic profile than hypertension with elevation in both systolic and diastolic blood pressure [44]. It is unknown if, in hemodialysis patients, the response of diastolic blood pressure to hypervolemia and to blood volume reduction by ultrafiltration is related to the hemodynamic profile of each patient. Moreover, it is unknown if their alterations in systolic blood pressure may be related to a different interaction between noradrenaline and angiotensin II than the alterations in diastolic blood pressure [45]. Our results underline the importance of including the assessment of Angiotensin II and its interaction with the ANS in future studies aimed at understanding the underlying mechanisms of blood pressure modulation during hemodialysis.

### Study Limitations and Perspectives

As limitations of our work, we had a relatively small sample, which limited the number of variables that could be adjusted in the multivariate analysis. Nevertheless, we were able confirm the association between angiotensin-II and HRV indices by adjusting for potential confounders such as age (Table 3). The included patients are heterogeneous in age, ESRD etiology and other characteristics, which reflect the usual heterogeneity of ESRD patients treated with hemodialysis worldwide. However, larger and more heterogeneous samples are needed to extend generalization of the present findings to the HD population. Notwithstanding, the internal validity of the study was strengthened by several aspects of the study: (i) we included patients not using anti-hypertensive drugs and without decompensated lungs or heart disease; (ii) several steps were applied to increase homogeneity in the hemodialysis prescription (such as a fixed total ultrafiltration rate and total ultrafiltration volume); (iii) all procedures to obtain the study variables (clinical information, blood samples, electrocardiographic recording) were performed with methods previously validated by our group with ample experience in such procedures.

Future studies are required to assess the potential clinical impact of RAAS and its interaction with the ANS during hemodialysis, including the risk of intradialytic hypotension, intradialytic hypertension and other relevant outcomes.

## 5. Conclusions

The results demonstrated that there were correlations between HRV indices and serum angiotensin II levels during HD. These results provide us with the first evidence of the co-activation of the RAAS with the ANS in HD patients and are the basis for further investigation of the physiological response to HD as well as the search for prognostic markers to predict intradialytic cardiovascular instability (such as hypotension and arterial hypertension).

## Figures and Tables

**Table 1:** Hemodynamic, biochemical and HRV variables evaluated in 20 patients during three moments of hemodialysis. Data are reported as the mean ± standard deviation or as the median (25th–75th percentile).

Variable	Intrahemodialysis Time		
	0.0 h	1.5 h	3.0 h
Systolic blood pressure (mmHg)	134 ± 18	127 ± 18	127 ± 28
Diastolic blood pressure (mmHg)	75 ± 13	75 ± 11	73 ± 22
Heart rate (bmp)	80 ± 13	71 ± 11 *	75 ± 12
Central temperature (°C)	33.3 ± 1.2	33.2 ± 1.2	33.1 ± 1.5
Peripheral temperature (°C)	27.6 ± 4.1	26.8 ± 4.2	26 ± 3.1
Log10 (angiotensin II) (pg/mL)	1.21 ± 0.38	1.22 ± 0.40	1.21 ± 0.38
Log10 (angiotensin 1–7) (pg/mL)	1.31 ± 0.23	1.30 ± 0.24	1.29 ± 0.25
Nitric oxide (pmol/mL)	80.38 ± 38.85	74.84 ± 36.92	77.41 ± 37.77
Total antioxidant capacity (mmol/L)	78.02 ± 36.59	69.38 ± 30.46	74.62 ± 34.68
Log10 (VLF) ms^2^	2.68 ± 0.40	2.94 ± 0.49 *	2.90 ± 0.46 *
Normalized VLF	0.59 ± 0.16	0.56 ± 0.18	0.64 ± 0.17
Log10 (LF) ms^2^	2.39 ± 0.57	2.65 ± 0.67 *	2.49 ± 0.72
Normalized LF	0.31 ± 0.13	0.31 ± 0.16	0.28 ± 0.13
Log10 (HF) ms^2^	1.82 ± 0.64	2.15 ± 0.86 *	1.91 ± 0.85 ^&^
Normalized HF	0.10 ± 0.08	0.13 ± 0.10	0.09 ± 0.07 ^&^
Log10 (LF/HF)	0.57 ± 0.33	0.50 ± 0.47	0.59 ± 0.33

* *p* < 0.05 vs. 0.0 h; ^&^ *p* < 0.05 vs. 1.5 h.

**Table 2:** Pearson’s correlation of serum levels of angiotensin II, angiotensin 1–7, nitric oxide (NO) and total antioxidant capacity. The correlation was calculated with 60 samples obtained from 20 patients with end-stage renal disease (ESRD) during hemodialysis. For each patient, blood pressure, heart rate, temperature and the serum levels of all molecules were obtained from measurements at 0, 1.5, and 3.0 h during hemodialysis.

Variable		Log10 (Angiotensin II) (pmol/mL)	Log10 (Angiotensin 1–7) (pmol/mL)	Nitric Oxide (pmol/mL)	Total Antioxidant Capacity (mmol/L)
	Mean ± SD	1.21 ± 0.38	1.30 ± 0.24	77.5 ± 37.8	74 ± 33.9
Age (years)	37 ± 12	−0.351 **	0.180	0.478 **	0.050
Body mass index (Kg/m^2^)	23.3 ± 3.1	−0.001	−0.115	0.155	−0.002
Systolic blood pressure (mmHg)	129 ± 22	0.123	−0.031	−0.253	−0.157
Diastolic blood pressure (mmHg)	74 ± 16	0.251	−0.136	−0.393 **	−0.335 **
Heart rate (bpm)	75 ± 13	0.212	−0.116	−0.259 *	0.021
Core temperature (°C)	33.2 ± 1.3	0.354 **	−0.118	−0.339 **	−0.314 *
Peripheral temperature (°C)	26.8 ± 3.9	−0.020	−0.039	−0.049	−0.121
Temperature difference (°C)	6.4 ± 3.7	0.140	0.001	−0.067	0.016
Log10 (VLF) ms^2^	2.84 ± 0.46	−0.071	−0.079	−0.040	−0.192
Normalized VLF	0.59 ± 0.17	0.390 **	0.038	−0.149	−0.116
Log10 (LF) ms^2^	2.51 ± 0.66	−0.229	0.148	0.037	0.097
Normalized LF	0.30 ± 0.14	−0.262 *	−0.140	0.138	0.063
log10 (HF) ms^2^	1.96 ± 0.79	−0.363 *	0.054	0.133	−0.010
Normalized HF	0.11 ± 0.08	−0.383 **	0.155	0.083	0.138
Log10 (LF/HF)	0.55 ± 0.038	0.359 **	−0.367 **	−0.213	−0.146

* *p*-value < 0.05; ** *p*-value < 0.01.

**Table 3:** Linear multiple regression analysis with predicted HRV indices and as independent variables, log_10_ angiotensin II (pmol/mL), age (years), diastolic blood pressure (mmHg), heart rate (beats per minute) and central temperature (°C). The regressions were calculated with 60 samples obtained from 20 patients with end-stage renal disease (ESRD) during hemodialysis. For each patient, blood pressure, heart rate, temperature, angiotensin and HRV indices were measured at 0, 1.5, and 3.0 h during hemodialysis.

Variables	Standardized β	β (C.I._95%_)	*p*	R^2^
**Predicted HRV Index: Normalized VLF**				0.574
Log_10_ (angiotensin II) (pmol/mL)	0.531	−0.244 (0.127–0.361)	<0.001	
Age (years)	0.438	0.006 (0.003–0.010)	0.001	
Diastolic blood pressure (mmHg)	0.070	0.001 (−0.002–0.003)	0.565	
Heart rate (bpm)	0.245	0.003 (0.001–0.007)	0.044	
Core temperature (°C)	−0.165	−0.022 (−0.055–0.010)	0.173	
***Predicted HRV Index:*** **Normalized LF**				0.167
Log_10_ (angiotensin II) (pmol/mL)	−0.351	−0.128 (−0.232–−0.024)	0.016	
Age (years)	−0.317	−0.004 (−0.007–0.001)	0.032	
Diastolic blood pressure (mmHg)	−0.166	−0.001 (−0.004–0.001)	0.222	
Heart rate (bpm)	−0.151	−0.002 (−0.005–0.001)	0.262	
Core temperature (°C)	0.150	0.016 (−0.013–0.045)	0.265	
***Predicted HRV Index:*** **Normalized HF**				0.340
Log_10_ (angiotensin II) (pmol/mL)	−0.531	−0.116 (−0.171–−0.061)	<0.001	
Age (years)	−0.393	−0.003 (−0.004–−0.001)	0.003	
Diastolic blood pressure (mmHg)	0.131	0.001 (−0.001–0.002)	0.278	
Heart rate (bpm)	−0.265	−0.002 (−0.003–0.001)	0.029	
Core temperature (°C)	0.096	0.006 (−0.009–0.021)	0.420	
***Predicted HRV Index:*** **log_10_ (LF/HF)**				0.263
Log_10_ (angiotensin II) (pmol/mL)	0.468	0.472 (0.202–0.742)	0.001	
Age (years)	0.247	0.008 (−0.001–0.016)	0.074	
Diastolic blood pressure (mmHg)	−0.164	−0.004 (−0.010–0.002)	0.202	
Heart rate (bpm)	0.258	0.008 (0.001–0.015)	0.044	
Core temperature (°C)	−0.105	−0.031 (−0.105–0.043)	0.405	

## Data Availability

The data presented in this study are available upon request from the corresponding author.
